# Single posterior debridement, interbody fusion, and fixation on patients with continuous multivertebral lumbar spine tuberculosis (CMLSTB)

**DOI:** 10.1186/s12891-020-03628-0

**Published:** 2020-09-10

**Authors:** Weihong Long, Liqun Gong, Yaqing Cui, Jie Qi, Dapeng Duan, Weiwei Li

**Affiliations:** Department of Orthopedic, Shaanxi Provincial People’s Hospital, Xi’an, 710068 Shaanxi China

## Abstract

**Background:**

Patients with continuous multi-vertebral lumbar spine tuberculosis (CMLSTB) were subjected to single posterior debridement, interbody fusion, and fixation to explore their clinical outcomes.

**Methods:**

Sixty-seven CMLSTB patients who underwent single posterior debridement interbody fusion and fixation between January 2008 to December 2017 were studied. The operation time, blood loss, perioperative complication rate, cure rate, Visual Analog Scale (VAS), Oswetry disability index (ODI), Japanese Orthopedic Association (JOA), Erythrocyte Sedimentation Rate (ESR), C-reactive protein (CRP), kyphotic Cobb’s angle and time of interbody fusion were analyzed to understand their therapeutic effects on CMLSTB patients.

**Results:**

The patients were followed up for 20–48 months, with a mean of 24.3 months. The mean operation time was 215.5 min (range, 120–280 min), whereas 818.0 ml of blood was lost (range, 400–1500 ml) with a perioperative complication rate of 6.0% and a cure rate of 95.5%. During the last phase of follow-up, the mean preoperative VAS score (5.7) and ODI (72.0%) decreased significantly to 1.4 (*t* = 31.4, *P*<0.01) and 8.4% (*t* = 48.4, *P*<0.01), respectively. Alternatively, the mean preoperative ESR and CRP (74.7 mm /h and 69.3 mg/L, respectively) decreased to average values (*t*_ESR_ = 39.7, *P*_ESR_<0.001; *t*_CRP_ = 50.2, *P*_CRP_<0.001), while the JOA score (13.9) significantly increased to 23.0 (*t* = − 11.6, *P*<0.01). The preoperative kyphotic Cobb’s angle (20.5°) decreased to 4.8° after the operation (*t* = 14.0, *P*<0.01); however, the kyphotic correction remained intact at the time of follow-up (*t* = − 0.476, *P = 0.635*). Furthermore, the mean of interbody fusion time was identified to be 8.8 months (range, 6–16 months).

**Conclusion:**

Single posterior debridement, interbody fusion, and fixation may be one of the surgical choices for the treatment of CMLSTB patients.

## Background

Spinal tuberculosis (TB), one of the most common forms of extrapulmonary TB, also known as Pott’s disease, is prevalent among the older population and mainly derived from active or latent pulmonary TB. It is characterized by the destruction of vertebral body that affect the load-bearing function of the body and cause symptoms such as durative back pain, fatigue, kyphotic deformity, or paralysis [1]. Spinal TB is associated with approximately half of all the cases that involve bone and joint TB in an impoverished population [2]. Recently, there has been an alarming increase in the incidence of extrapulmonary TB in people from developing countries, which became a substantial economic burden on the families of the affected and the entire society. Most of the spinal TB lesions are limited to 1–2 vertebrae; however, due to lack of timely treatment or rapid progression of the disease, spinal TB lesions can be observed in three or more vertebras. Such patients with three or more consecutive affected vertebras suffer from continuous multi-vertebral spinal TB (CMSTB) are always challenging to treat due to the poor nutritional status and great chance of other concurrent extrapulmonary TB lesion [3]. Though chemotherapy is the main line of care for patients with spinal TB; however, surgical intervention is necessary for patients with vertebral collapse, acute abscess, sinus tract, dead bone, severe kyphosis deformity and neurological deficits [4]. The surgical methods for spinal TB include single anterior debridement interbody fusion and fixation, single posterior debridement interbody fusion and fixation, and combined posterior pedicle screw fixation with one or two stages of anterior debridement interbody fusion. The single anterior debridement interbody fusion and fixation technique can access lesion area easily, which is beneficial for debridement and reconstruction of the anterior vertebral column; however, it has some inherent drawbacks, such as the risk of visceral and vascular injury, feeble strength of fixation [5]. Combined posterior pedicle screw fixation with one or two stages of anterior debridement interbody fusion can effectively remove the lesion to provide excellent anterior column support and robust posterior instrumentation; nevertheless, it is always accompanied by long operation time, extensive blood loss and high complications related to trauma [6]. The single posterior debridement interbody fusion and fixation technique is suitable for spinal TB patients with posterior column destruction of the spine. However, there have been reports of patients with mono-segmental spinal TB who underwent a single posterior approach with excellent clinical results [7]. The current study aims to evaluate the clinical efficacy and feasibility of single posterior debridement interbody fusion and fixation for patients with continuous multivertebral lumbar spine TB (CMLSTB).

## Methods

This study included 67 CMLSTB patients consisting of 28 females and 39 males, who were treated surgically with the single posterior debridement interbody fusion, and fixation technique between January 2008 to December 2017. The ethics review committee of Shaanxi Provincial People’s Hospital approved this study, and informed consent was obtained from all patients before undergoing the technique. The initial diagnosis was made through clinical presentations, laboratory indexes, and imaging manifestations, which was finally confirmed by mycobacterium TB (MTB) culture or histopathological performance of caseous necrosis and typical granuloma. The mean age of the subjects was 51.0 ± 13.0 years, which ranged between 22 to 72 years with a mean duration of disease before definite diagnosis to be 14.5 ± 5.8 months (range, 6–28 months) (Table 1). There were 37 cases with three affected vertebras, 21 cases with four affected vertebras, and 9 cases with five or more affected vertebras. All the patients presented with severe or mild low back pain, 39 of which were also symptomatic with fever, sweating, emaciation, and other symptoms of TB toxicity. Among the cases, 18 patients presented with a radiating ache of the unilateral lower extremity, while 34 cases also had simultaneous obvious neurologic deficits (Table 1). The means of the preoperative Visual Analog Scale (VAS), preoperative Oswestry disability index (ODI) and preoperative Japanese Orthopedic Association (JOA) scores were 5.7 ± 1.0 (range, 4–8), 72.0 ± 9.9 (range, 50–84%) and 13.9 ± 5.6 (range, 6–26), respectively (Table 2). The mean preoperative Erythrocyte Sedimentation Rate (ESR) and C-reactive protein (CRP) value were 74.7 ± 12.3 mm/h (range, 42–108 mm/h) and 69.3 ± 10.9 (50–103 mg/L), respectively. The mean preoperative kyphotic Cobb’s angle was 20.5° ± 7.0° (range, 0°-32°) (Table 3).

**Table 1:** Clinical data of patients

Parameters	mean ± SD or NO/%
Average age (years)	51.0 ± 13.0
Sex ratio (male/total)	39:67 (58.2%)
Mean of BMI (kg/m^2^)	18.8 ± 3.6
Duration of disease before definite diagnosis (months)	14.5 ± 5.8
Anemia	54 (80.6%)
Hypoproteinemia	49 (73.1%)
Concurrent extra-pulmonary TB	7 (10.4%)
Clinical symptoms	
TB toxic symptoms	39 (58.2%)
Back pain	67 (100%)
Lower limb radiating ache	18 (26.9%)
Neurological deficit	34 (50.7%)
Number of affected vertebras	
3 vertebras	37 (55.2%)
4 vertebras	21 (31.3%)
5 or more than 5 vertebras	9 (13.4%)
Abscess	
None	0
Small amount	49
Large amount	18

**Table 2:** Comparison for VAS, ODI and JOA value in preoperative, postoperative and final follow up

Schedule	VAS	ODI(%)	JOA
Pre-op	5.7 ± 1.0* (*t* = −31.4, *P* < 0.01)	72.0 ± 9.9* (*t* = −48.4, *P* < 0.01)	13.9 ± 5.6* (*t* = 11.6, *P* < 0.01)
Post-op	3.2 ± 0.6# (*t* = 17.3, *P* < 0.01)	25.2 ± 11.5# (*t* = 25.2, *P* < 0.01)	18.7 ± 2.9# (*t* = −6.25, *P* < 0.01)
FFU	1.4 ± 0.4Δ (*t* = 19.2, *P* < 0.01)	8.4 ± 4.1Δ (*t* = 11.3, *P* < 0.01)	23.0 ± 3.1Δ (*t* = −8.28, *P* < 0.01)

*FFU* Final follow up

*: *P* < 0.01, compared with FFU indexes

#: *P* < 0.01, compared with pre-op indexes

Δ: *P* < 0.01, compared with post-op indexes

**Table 3:** Comparison for ESR, CRP and kyphosis angle in preoperative, postoperative and final follow up

Schedule	ESR	CRP	Kyphosis angle(°)
Pre-op	74.7 ± 12.3* (*t* = −39.7, *P* < 0.01)	69.3 ± 10.9* (*t* = −50.2, *P* < 0.01)	20.5 ± 7.0* (*t* = −13.2, *P* < 0.01)
Post-op	34.0 ± 9.7# (*t* = 21.2, *P* < 0.01)	20.9 ± 6.4# (*t* = 31.3, *P* < 0.01)	4.8 ± 6.0# (*t* = 14.0, *P* < 0.01)
FFU	13.7 ± 2.6Δ (*t* = 16.6, *P* < 0.01)	2.2 ± 0.9Δ (*t* = 23.5, *P* < 0.01)	5.3 ± 6.3 (*t* = − 0.476, *P* = 0.635)

*FFU* Final follow up

*: *P* < 0.01, compared with FFU indexes

#: *P* < 0.01, compared with pre-op indexes

Δ: *P* < 0.01, compared with post-op indexes

### Preoperational protocol

The chemotherapeutic regime that was followed consisted of injecting isoniazid (intravenous drip, 300 mg per day), rifampin (oral, 450 mg per day), pyrazinamide (oral, 750 mg per day), ethambutol (oral, 750 mg per day), and levofloxacin (intravenous drip, 0.4 g, per day). The values of ESR and CRP were recorded every 3 days before the operation. Anti-TB chemotherapy lasted for 2 weeks until a dramatic decrease in their values was observed. Anemia and hypoproteinemia were corrected before the operation, and nutritional support was provided to patients (along with painkillers), which involved minimal overactive movement out of bed.

### Procedure

**Fig. 1:**
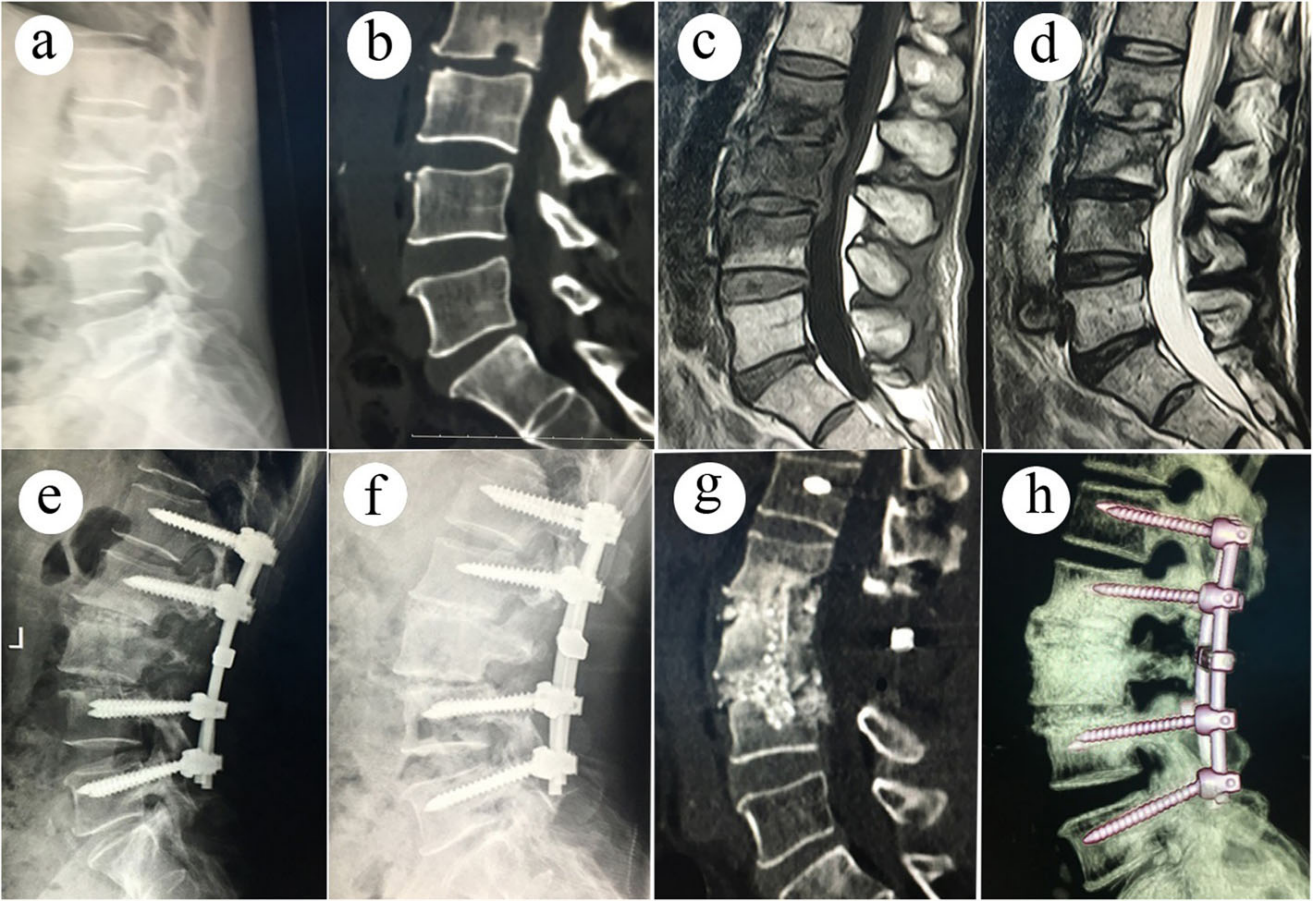
A 53-year-old male patient with L2–4 spinal tuberculosis received single posterior debridement, interbody fusion, and fixation. (**a-d**). Patients given standard anti-TB therapy showed chronic aggravation of disc and bone destruction in L2–4: (**e**) Postoperative images after 10 days of operation showed good position of pedicle screw fixation. (**f-g**) Postoperative images after 24 months of operation showed that solid interbody fusion with no displacement, breakage of instrumentation, and insignificant loss of the kyphotic correction

**Fig. 2:**
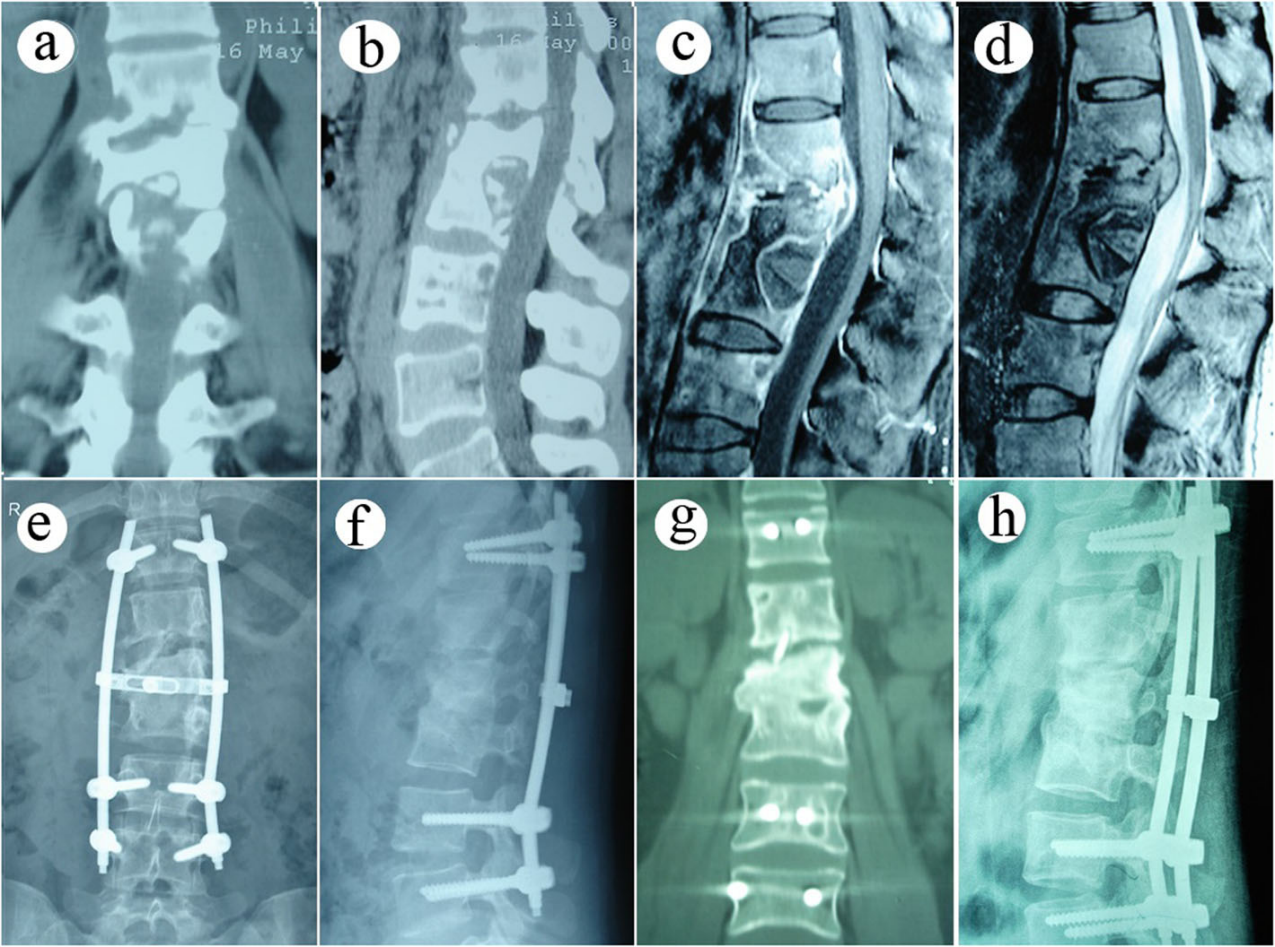
A 48-year-old female patient with T12-L3 spinal tuberculosis received single posterior debridement, interbody fusion, and fixation. (**a–d**). Preoperative CT and MRI images showed dead bone formation, bone and discs damage, and paravertebral abscess formation in T12-L3. (**e-f**) Postoperative image after 7 days of operation showed the pedicle screw fixation across the focal area was appropriately situated (**g-h**) Postoperative images after 28 months of operation showed that solid interbody fusion was obtained with insignificant loss of the kyphotic correction

A longitudinal incision was made along the spinous process after adopting the prone position to dissect out the paravertebral muscle until a good view of the lamina and bilateral facet joints was achieved. Pedicle screws were placed in vertebrae that were adjacent to the diseased vertebras. Pedicle screws were also placed in the diseased vertebrae when the upper 1/2 of the vertebral body was not severely destroyed. A temporary short rod was placed to stabilize the surgical zone. Full lamina was removed for canal decompression, acquisition of ample space for intervertebral debridement, and acquirement of bone graft materials. Tools, such as various angles curettes, reamers, and scrapers, were used to clean up the necrotic discs, dead bones, and caseous granulomatous. Diseased sclerotic bone was also removed for radical debridement and the establishment of a suitable bone graft zone. Paravertebral abscesses were drained by placing a catheter in the indirect sight lesion for high-speed lavage through the intervertebral space. The autogenous iliac block obtained from the posterior superior iliac spine was sheared and placed in the intervertebral space for interbody fusion. The permanent rod was curved and placed bilaterally. Intertransverse fusion was carried out with the residual bone graft material using 1 g rifampicin powder. The dissected diseased tissue was sent for histopathological observation and microorganism culture. (Figs. 1 and  2)

### Postoperative management

Prophylactic antibiotic therapy was given to patients for 24 h, and the drainage tube was removed once the volume of drainage was less than 20 ml per 24 h. The patients were advised to ambulate slightly under the protection of orthosis after drainage removal. The protection of orthosis was maintained until interbody fusion was done, and the activity of walking was slowly and gradually increased. Standard quadruple anti-TB drug therapy was continued for 18–20 months, and the liver function was monitored for the duration of the prophylactic drug treatment.

### Evaluation of indexes and follow-ups

The operation duration, blood loss, perioperative complication, and cure rate were recorded. Patients were followed up at an interval of three-month within the first year of operation. Subsequent follow up was done every 6 months within the second year of surgery and once every year after that. Indexes such as the VAS score, ODI score, JOA score, ESR, CRP, kyphotic Cobb’s angle, and time of interbody fusion were updated during the follow-up visits. The interbody fusion was confirmed by the presence of trabecular bone bridging between the bone graft and adjacent vertebras on an X-ray or CT scan.

### Statistics

SPSS 22.0 (SPSS company, USA) statistical software was used for the data analysis. The data of evaluative indexes was expressed as mean ± SD. A paired samples test was used for comparison of indexes before and after the operation. *P* value of less than 0.05 was considered to be significantly significant.

## Results

Sixty-seven patients were followed-up for a period ranging from 20 to 48 months (mean 24.3 months). The mean duration of operation was 215.5 ± 38.7 min (range, 120–280 min), and the mean blood loss was 818.0 ± 181.7 ml (range, 400–1500 ml) (Table 4). Out of 67 patients, two cases had complications from dura tear and leakage of cerebrospinal fluid, while one had calf muscular venous thrombosis, and the other case had fat liquefaction at the site of the wound. The perioperative complication rate was 6.0% (Table 4). Three cases had unsuccessful outcomes for single posterior debridement interbody fusion and fixation, of which two had revision surgeries for anterior open debridement and pus drainage, which ultimately healed by the following up period. The cure rate was 95.5%. The mean postoperative VAS score was 3.2 ± 0.6, which was significantly lower than the preoperative one (*t* = 17.3, *P*<0.01) and decreased to 1.4 ± 0.4 by the follow-up period (*t* = 19.2, *P*<0.01) (Table 2). Likewise, the mean postoperative ODI score was 25.2 ± 11.5%, which was significantly lower than the preoperative one (*t* = 25.2, *P*<0.01) and decreased to 8.4 ± 4.1% by the follow-up period (*t* = 11.3, *P*<0.01) (Table 2). The mean postoperative JOA score was 18.7 ± 2.9, which was significantly higher than the preoperative one (*t* = − 6.25, *P*<0.01) and increased to 23.0 ± 3.1 in the final follow up (*t* = − 8.28, *P*<0.01) (Table 2). Similarly, the mean postoperative ESR (34.0 ± 9.7 mm/h) and CRP (20.9 ± 6.4 mg/L) values was significantly lower than the preoperative one (*t*_ESR_ = 21.2, *P*_ESR_<0.01; *t*_CRP_ = 31.3, *P*_CRP_ <0.01) and declined to 13.7 ± 2.6 mm/h and 2.2 ± 0.9 mg/L, respectively (*t*_*ESR*_ = 16.6, *P*_ESR_<0.01; *t*_CRP_ = 23.5, *P*_CRP_<0.01) (Table 3). The kyphotic Cobb’s angle of 4.8° ± 6.0° was significantly lower than the preoperative one (*t* = *14.0, P*<0.01), and increased to 5.3° ± 6.3° (*t* = − 0.476, *P = 0.635*) invisibly (Table 3). The mean interbody fusion time was 8.8 ± 1.9 months (range, 6–16 months).

**Table 4:** Items related to surgery

Items related to surgery	mean ± SD or NO/%
Operation time (min)	215.5 ± 38.7
Blood loss (ml)	818.0 ± 181.7
Complication	4 (6.0%)
Cerebrospinal fluid leakage	2 (3.0%)
Calf muscular venous thrombosis	1 (1.5%)
Wound fat liquefaction	1 (1.5%)
Failure for first surgery and accept anterior revision surgery	3 (4.5%)
Percutaneous lavage and drainage under CT guide	1 (1.5%)
Small incision debridement	1 (1.5%)
Open extensive debridement	1 (1.5%)

## Discussions

The detection of spinal TB in patients is difficult as the course of the disease is usually slow. In the early stage of spinal TB, the patients experience pain at the lesion site without any particular manifestations, which delay its appropriate diagnosis. The lesion site occurs in the lumbar, followed by the thoracic and cervical due to its specific weight-bearing functionality. The lumbar region is categorized into central and marginal types based on X-ray features. The central type constitutes of skeleton collapse, wherein the vertebral body is destroyed by induced mycobacterium TB via Baston’s venous plexus. The marginal type occurs as disc necrosis, endplate erosion, and subchondral-bone destruction [8, 9]. CMLSTB patients are diagnosed with both central and marginal types of lumber disorder, which are prevalent in elderly and frail patients taking immunosuppressants for an extended period. They have severe TB-toxic symptoms, such as fever, night sweats, loss of appetite, and weight loss. Due to a damaged spine, they suffer from severe spinal instability or kyphosis deformity. Moreover, few patients also have paraplegia; and therefore, the treatment for CMLSTB patients is a challenge for spine surgeons [10]. Preoperative chemotherapy is essential for the effective management of CMLSTB patients [11]. The operation time was selected based on the following factors: standard quadruple anti-TB drugs for 2 to 4 weeks, followed by the second-line anti-TB drugs if necessary; improved condition of the patients’ physical condition and immunity, along with rectification of their anemia and hypoproteinemia and a significant decrease of their ESR index below 40 mm /h. Surgery was only recommended after adequate measures were taken to control systematic TB disease. Furthermore, the sensitivity of chemotherapy was also evaluated. In the current study, all the patients received 2 weeks of the first-line anti-TB drug treatment. Among these, 14 patients received 2 weeks of levofloxacin and an additional 2 weeks of first-line anti-TB therapy due to a non-significant decrease of their ESR and CRP indexes. The purpose of surgery in spinal TB is to cure the TB lesions, reconstruct spinal stability, and prevent neurological damage [12]. The observed surgical indications for spinal TB are apparent cold abscess, sinus tract formation, prominent dead bones or cavities in the vertebral body, and spinal cord compression. The current standard of surgical care for the spinal TB patients includes single anterior debridement interbody fusion and fixation, combined posterior pedicle screw fixation with one or two-stage anterior debridement interbody fusion and single posterior debridement interbody fusion and fixation [13, 14]. The surgical technique was selected based on many presenting symptoms and factors, such as the location of TB lesion, manner of bone destruction, degree and range of lesion, number of vertebral bodies involved, patient’s physical condition, and concomitant medical disorders [14, 15]. The process of single anterior debridement interbody fusion and fixation is developed from the Hong Kong procedure, as reported by Hodgson et al. in 1960 [16, 17]. Such a procedure effectively removes the lesion, eliminates interbody bone graft, and installs the fixation. However, the fixed vertebral support cannot tolerate the torsional force as its strength is weak. Consequently, the loss of kyphosis deformity correction was observed in recovered patients during the follow-up period. Furthermore, as the range of fixation is more than three vertebral bodies, the obstacle from the surrounding organs loosens the fixation [18]. The use of posterior pedicle screw fixation with one or two stages anterior debridement interbody fusion is reported to have an excellent therapeutic effect on spinal TB [19]. However, this procedure has limitations such as two incisions, longer operation time, blood loss, trauma, and a high risk of common infections for wound [20]. Pedicle screw fixation is suitable for spinal TB patients who developed lesions in the posterior column [21]. Although the procedure destroys the posterior structure of the spine, most scholars completed mono-segmental surgery for spinal TB with excellent clinical outcomes [22, 23]. In this study, we observed that single posterior debridement interbody fusion and fixation is useful for CMLSTB patients with a cure rate of 95.5%. Significant improvements were observed in postoperative indexes such as VAS score, ODI score, JOA score, the value of ESR, and CRP. Furthermore, we also observed that the perioperative complication rate was low (6.0%). Wu et al. [24] studied 62 cases of multisegment thoracic spinal TB in patients with kyphosis. Such patients received one-stage posterior surgery of debridement interbody fusion and instrumentation, which prevented TB recurrence during the follow-up duration. Cui et al. [25] reported in their retrospective study that they analyzed 81 patients with CMSTB, including 39 patients who underwent anterior transthoracic debridement titanium interbody fusion and fixation, posterior transcostotransverse decompression strut grafting and pedicle fixation, combined anterior debridement and strut grafting with posterior fixation. Cui et al. [25] observed that posterior fixation was more effective than anterior fixation for kyphosis correction; however, sinus formation was found to be associated with posterior debridement, interbody fusion, and fixation. They also evaluated the single posterior debridement, interbody fusion and fixation technique for lumbar CMSTB. Lumbar constitutes the lower part of the spine and bears the maximum weight, and hence, it is often associated with internal fixation failure [26]. Currently, there is no literature relevant to lumbar CMSTB. In our series, we observed that single posterior debridement interbody fusion and fixation could treat CMLSTB for several reasons. First, most patients were sensitive to anti-TB drug therapy; hence, regulation of anti-TB drug dosage was necessary before surgery. Second, surgery was only conducted on patients after significant improvements were observed in their nutritional status and immunity, none of these patients had a history of active TB as well. Third, the surgeons who performed the surgeries were skilled at performing posterior debridement interbody fusion and pedicle fixation on CMLSTB patients. Fourth, a lengthy and effective anti-TB treatment was recommended after surgery [27]. The current study had some inherent limitations. First, it is a retrospective study, and secondly, it does not have any control cases. Moreover, the number of the included cases is reported to be small, which affected many subjective factors, such as selection bias, and lack of suitable methods for immunity assessment, etc. Thus, the conclusion needs to be further confirmed by the multi-center prospective randomized controlled study.

## Conclusions

Single posterior debridement, interbody fusion, and fixation may be one of the surgical choices for the treatment of CMLSTB patients.

## Data Availability

The datasets supporting the conclusions of this article are included within the article. The raw data can be requested from the corresponding author on reasonable request.

## Abbreviations

TB: Tuberculosis
CMSTB: Continuous Multi-vertebral Spine TB
CMLSTB: Continuous Multi-vertebral Lumbar Spine TB
MTB: Mycobacterium TB
VAS: Visual Analog Scale
ODI: Oswetry disability index
JOA: Japanese Orthopedic Association
ESR: Erythrocyte Sedimentation Rate
CRP: C-reactive protein
